# Supplementary material for: International wildlife trade quotas are characterized by high compliance and coverage but insufficient adaptive management
Source: Nat Ecol Evol. 2024 Sep 9;8(11):2048–57. doi: 10.1038/s41559-024-02531-4 (PMC11540902; doi:10.1038/s41559-024-02531-4)
Supplement: Supplementary file 1 — Supplementary Figs. 1–8 and Tables 1–6. [file 41559_2024_2531_MOESM1_ESM.pdf]

nature ecology & evolution

Supplementary information https://doi.org/10.1038/s41559-024-02531-4

International wildlife trade quotas are characterized by high compliance and coverage but insufficient adaptive management

In the format provided by the authors and unedited

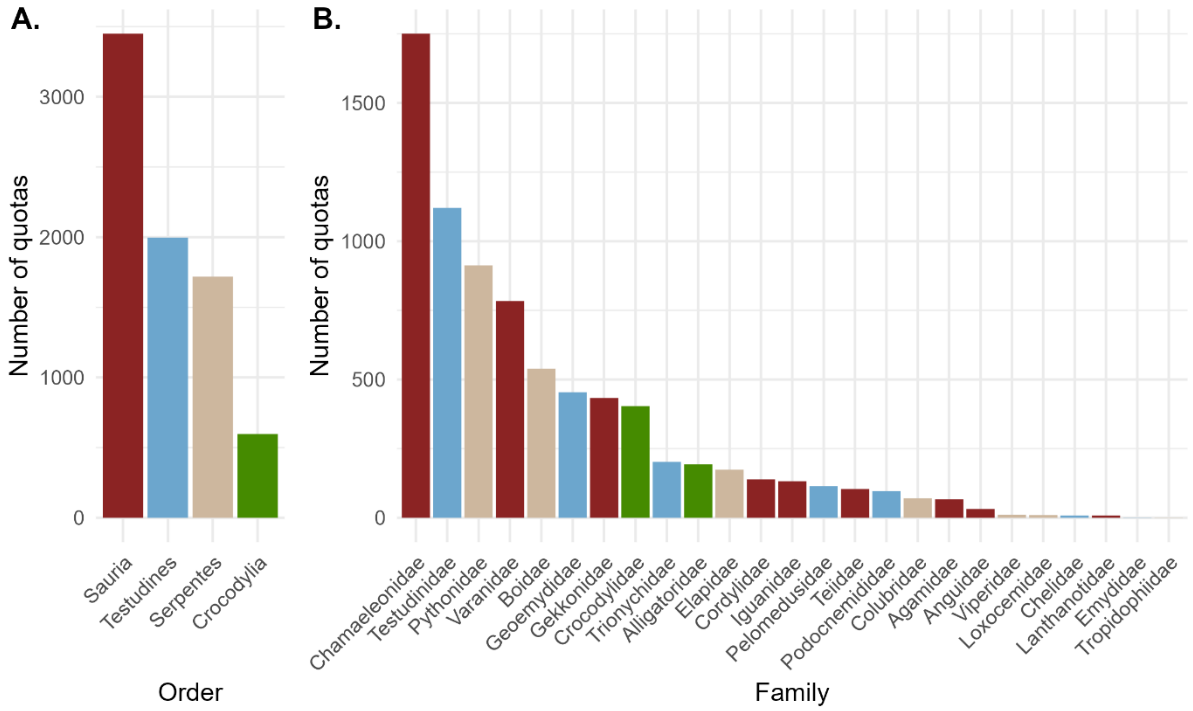

Supplementary Figure 1. Number of quotas across reptile taxa. Coloured bars represent the Orders shown in

panel A.

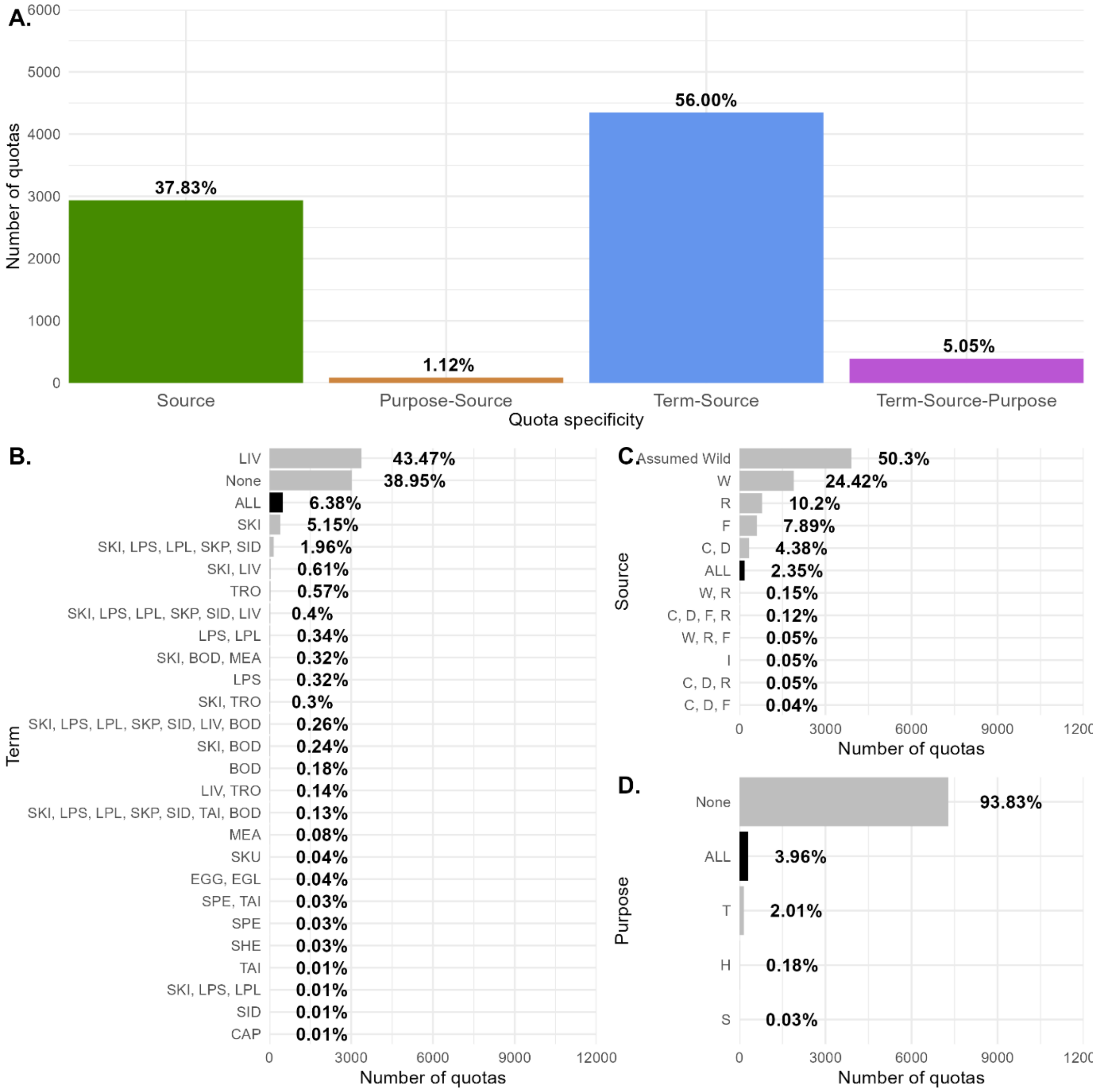

Supplementary Figure 2. Coverage breakdown for 7761 reptile quotas for the period 1997-2021. A. Level of detail specified in the quota notes. B. Specific terms specified as covered by the quota. C. Specific sources specified as covered by the quota. D. Specific purposes specified as covered by the quota. In all cases, the percentages denote the proportion of quotas falling into that group. In panels B, C and D the percentage of quotas stated to cover all terms, sources or purposes are shown in black. All term, source and purpose codes follow CITES designations (definitions can be found in Table S1).

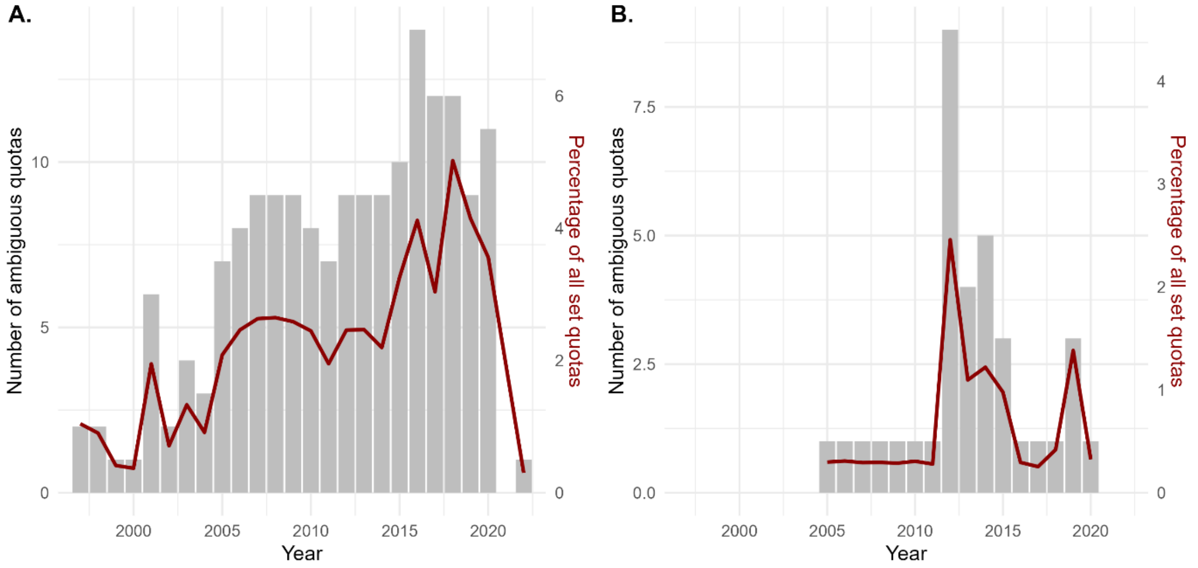

Supplementary Figure 3. Tally of quotas through time that specify ambiguous terms. A. Tally of quotas specifying both whole skins and derived skin products in the same quota. B. Tally of quotas specifying both live and derived skin products in the same quota. The grey bars denote the actual number of ambiguous quotas per year and correspond to the left y-axis. The red line denotes this number as a proportion of all reptile quotas set in that year and corresponds to the right y-axis.

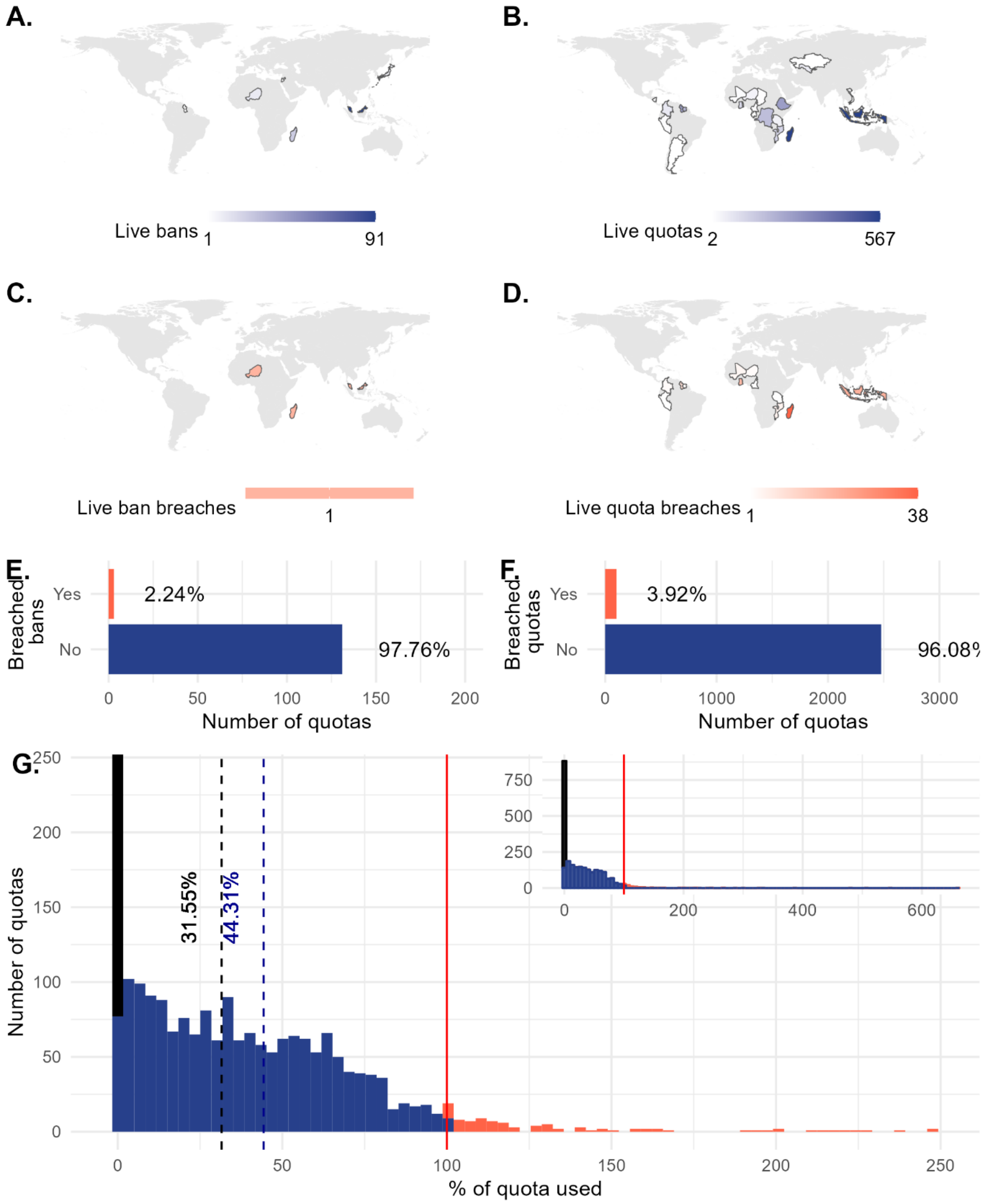

Supplementary Figure 4. Live reptile quota compliance 1997-2021, using importer reported data. Counts of total live A. trade bans (zero-quotas) and B. trade quotas. Counts of live C. trade ban breaches and D. quota breaches. Tallies of the total number of adhered to and breached E. bans and F. quotas. G. Percentage of each quota used (0% indicates the species had a quota but was not traded, 100% would indicate exactly the quota amount was traded and percentages above 100% indicate quota breaches). Dashed black line indicates the average percentage of a quota used including species never traded but under quota (e.g. including the 0% values), dashed blue line indicates the average percentage of a quota used for species that have been traded (e.g. excluding the 0% values) and the solid red line indicates 100% quota use. Note – the main panel x-axis

truncated at 250% quota use for clarity and the y-axis at 100 quotas, the full distribution is show in the inset panel. Only 20 quotas had a percentage use over 250%.

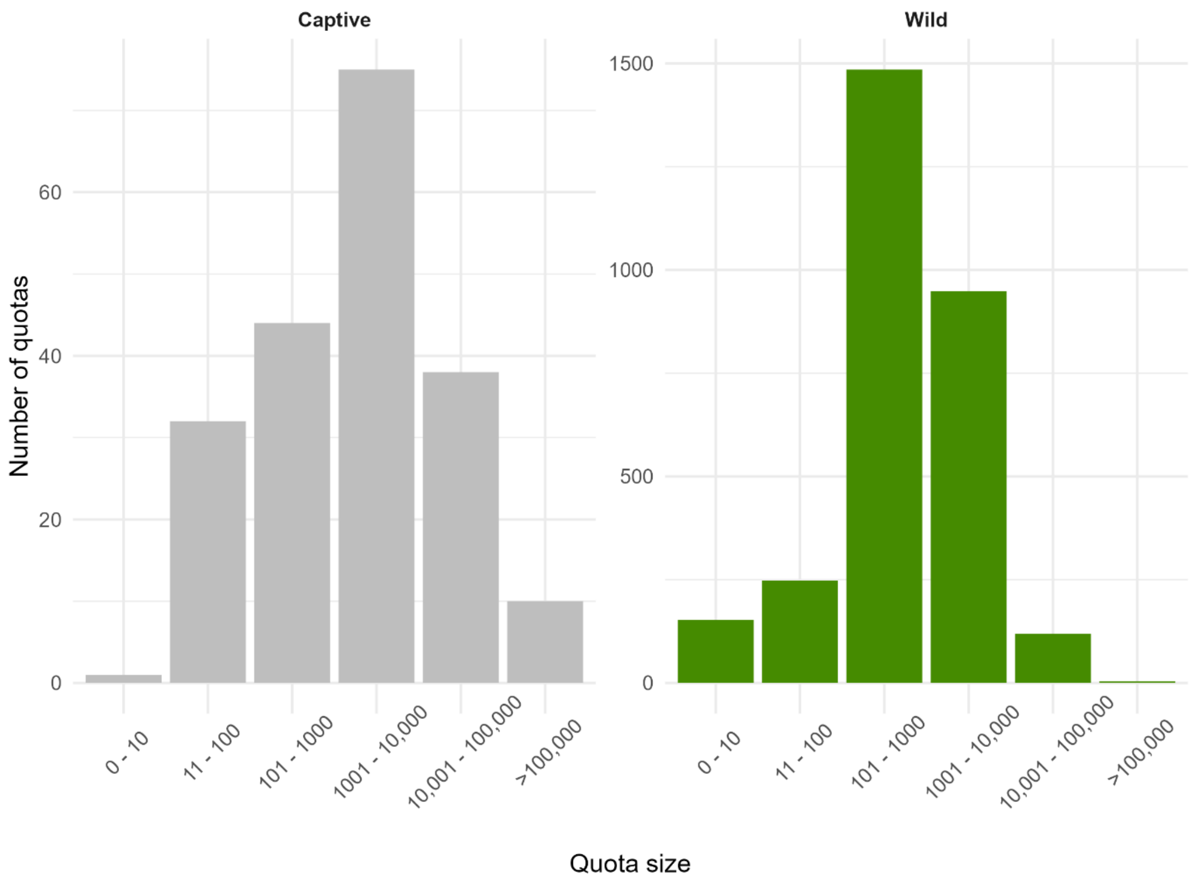

Supplementary Figure 5. Live quota sizes set for wild and captive specimens. Wild defined as source codes W (wild taken) and R (taken from the wild and ranched) and captive as codes C (captive bred in accordance with the conventions definitions), D (registered Appendix I listed captive breeding facility) and F (captive born, F1 generation or otherwise not in accordance with the Conventions definition of captive bred).

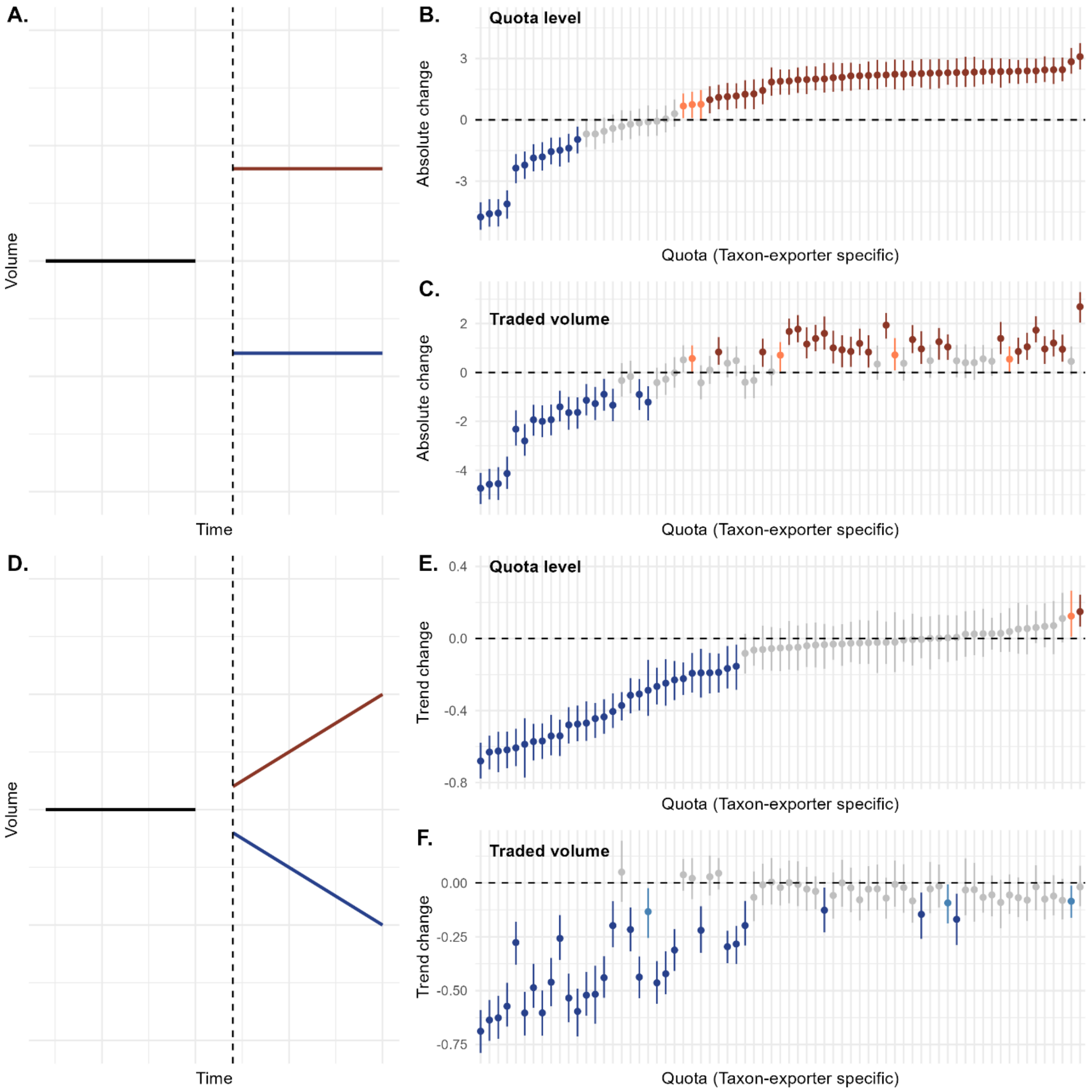

Supplementary Figure 6. Trade volume coefficients pre- to post-quota setting. A. Conceptual figure for an absolute increase or decrease in quota level or traded volume relative to pre-quota volumes. B. Counterfactual coefficients for the quota level relative to volumes the year prior to implementation (β_2.). A one denotes a one SD increase in quota levels relative to pre-quota traded volumes. C. As B. but for actual traded volumes (β_1). D. Conceptual figure for an increase or decrease in quota level or traded volume trends through time relative to pre-quota trends. E. Counterfactual coefficients for trend changes in quota level relative to volumes the year prior to implementation (β_5). Minus one denotes a -1 SD decrease in traded volumes per year with a quota relative to without. F. As E but for actual traded volumes (β_4). Colours in C, D, F and G denote the direction and certainty of difference: red (+*ve* and *pd* > 97.5%), orange (+*ve* and *pd* > 95.0%), grey (*pd* < 95.0%), pale blue (-*ve* and *pd* > 95.0%), and dark blue (-*ve* and *pd* > 97.5%). All points are medians and error bars the 90% highest density posterior intervals. Coefficients shown for each of the 69 distinct species-exporter time series, covering 12 Parties and 68 species.

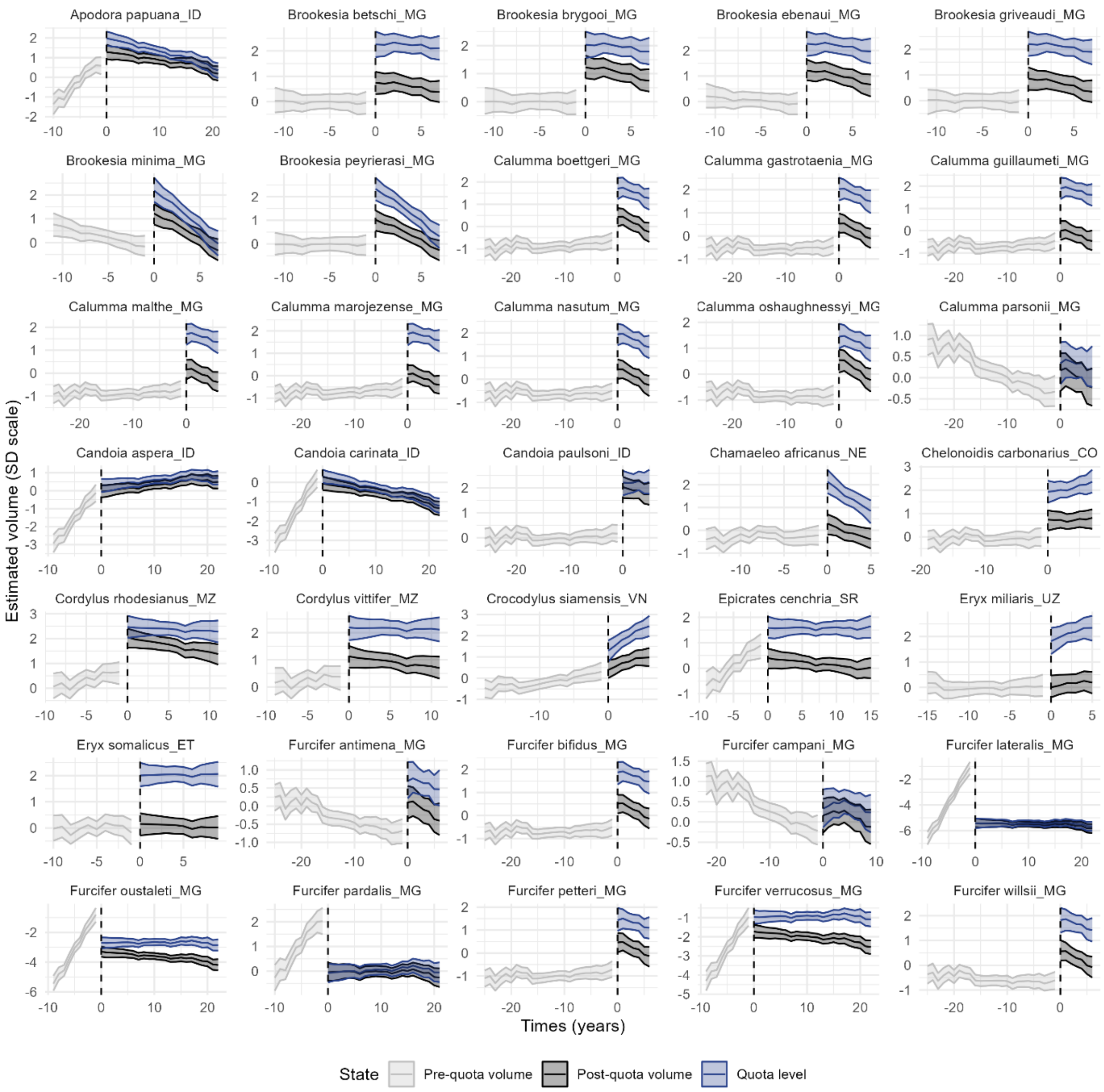

Supplementary Figure 7. Quota-specific estimates for pre and post-quota trade relative to quota levels (Part 1 – plots split over two figures for clarity). Plot titles denote the species and Party shown. Colours should pre-quota trade (grey), post-quota trade (volume) and quota levels (blue). The dashed black line at zero shows the point quotas were established. All lines are posterior medians and ribbons are 90% HDI's.

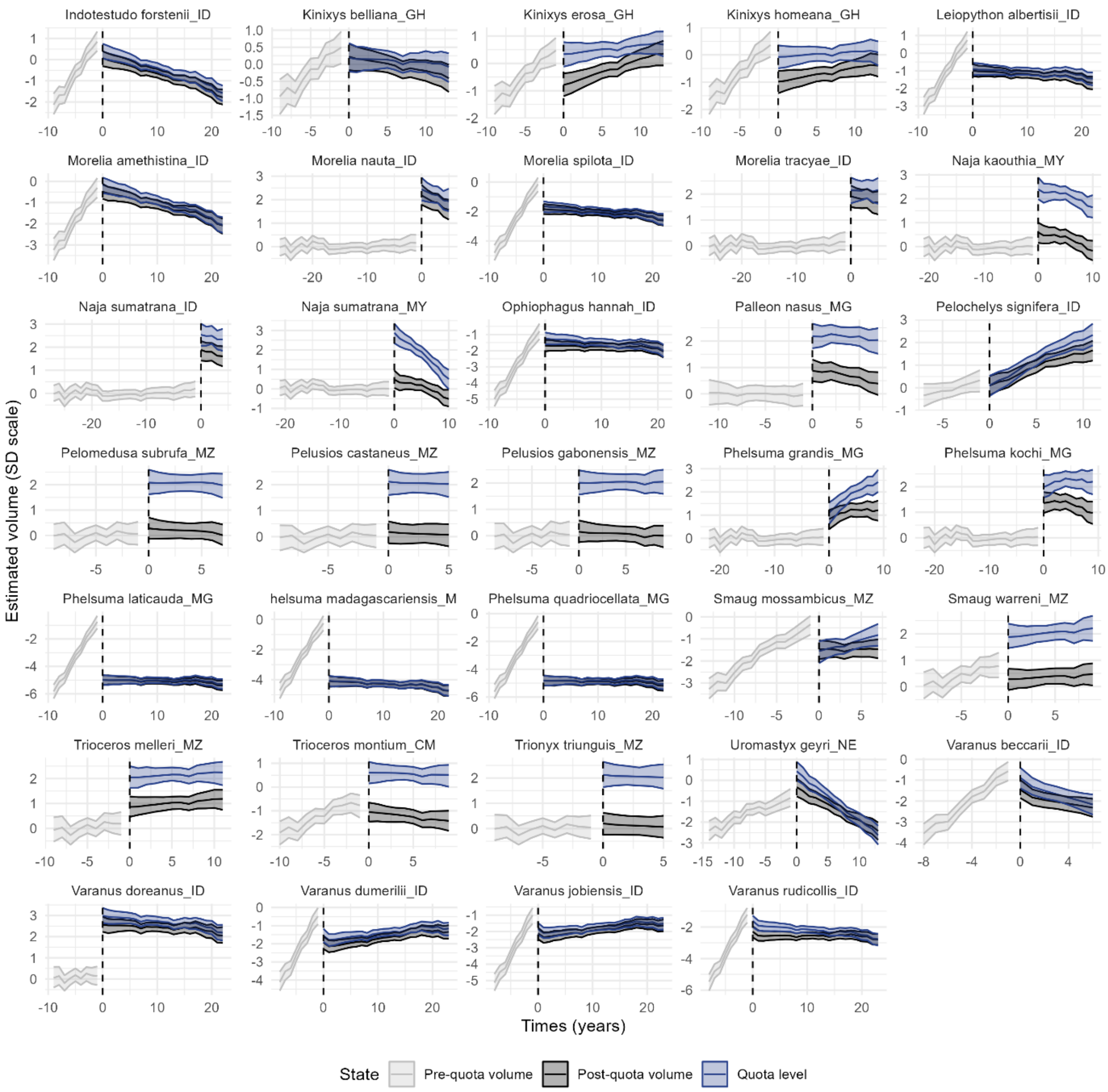

Supplementary Figure 8. Quota-specific estimates for pre and post-quota trade relative to quota levels (Part 2 – plots split over two figures for clarity). Plot titles denote the species and Party shown. Colours should pre-quota trade (grey), post-quota trade (volume) and quota levels (blue). The dashed black line at zero shows the point quotas were established. All lines are posterior medians and ribbons are 90% HDI's.

Supplementary Table 1. CITES summary codes used in Figure S1.

Type	Code	Interpretation
**Term**	LIV	Live
	SKI	Skin
	LPS	Leather product (small)
	LPL	Leather product (large)
	SKP	Skin piece
	SID	Side
	TRO	Trophy
	BOD	Body
	MEA	Meat
	TAI	Tail
	SKU	Skull
	SPE	Specimen (scientific)
	SHE	Shell
	CAP	Carapace
	EGG	Egg
	EGL	Egg (live)
**Source**	W	Taken from the wild
	R	Ranched. specimens of animals reared in a controlled environment, taken as eggs or juveniles from the wild, where they would otherwise have had a very low probability of surviving to adulthood.
	F	Animals born in captivity (F1 or subsequent generations) that do not fulfil the definition of ‘bred in captivity’ in Resolution Conf. 10.16 (Rev.), as well as parts and derivatives thereof.
	C	Animals bred in captivity in accordance with Resolution Conf. 10.16 (Rev.), as well as parts and derivatives thereof, exported under the provisions of Article VII, paragraph 5.
	D	Appendix-I animals bred in captivity for commercial purposes in operations included in the Secretariat’s Register, in accordance with Resolution Conf. 12.10 (Rev. CoP15).
	I	Confiscated or seized specimens
**Purpose**	T	Commercial
	H	Hunting
	S	Scientific

Supplementary Table 2. List of all quotas where trade was reported by the exporter and compliant and reported by the importer and in breach. Percentages shown are percentages of the set quota reported as traded in that year. Party codes are as follow, NE (Niger), MG (Madagascar), GH (Ghana), CM (Cameroon), MZ (Mozambique), ID (Indonesia), PE (Peru), SR (Suriname), GY (Guyana) and TZ (United Republic of Tanzania).

Taxon	Party	Year	Quota	ER [Volume (%)]	IR [Volume (%)]
Uromastyx geyri	NE	2008	0	0 (-)	200 (-)
Brookesia minima	MG	2017	0	0 (-)	21 (-)
Python regius	GH	2009	200	140 (70%)	1320 (660%)
Python regius	GH	2008	200	130 (65%)	960 (480%)
Python regius	GH	2011	200	200 (100%)	770 (385%)
Kinixys homeana	GH	2002	340	119 (35%)	1109 (326.2%)
Chamaeleo senegalensis	GH	2002	1500	1222 (81.5%)	3346 (223.1%)
Furcifer lateralis	MG	1999	2000	1806 (90.3%)	4398 (219.9%)
Python regius	GH	2010	200	50 (25%)	420 (210%)
Kinixys homeana	GH	2001	340	0 (0%)	683 (200.9%)
Trioceros quadricornis	CM	2001	400	50 (12.5%)	789 (197.2%)
Erymnochelys madagascariensis	MG	2001	25	0 (0%)	42 (168%)
Cordylus vittifer	MZ	2002	1000	1000 (100%)	1599 (159.9%)
Cuora amboinensis	ID	2004	18000	15700 (87.2%)	25577 (142.1%)
Amyda cartilaginea	ID	2009	25200	25200 (100%)	35308 (140.1%)
Amyda cartilaginea	ID	2008	25200	25197 (100%)	35230 (139.8%)
Siebenrockiella crassicollis	ID	2004	4500	3737 (83%)	6089 (135.3%)
Amyda cartilaginea	ID	2013	25200	25200 (100%)	33636 (133.5%)
Uroplatus lineatus	MG	2011	63	41 (65.1%)	83 (131.7%)
Iguana iguana	PE	1997	4000	3850 (96.2%)	5250 (131.2%)
Brookesia stumpffi	MG	2018	500	494 (98.8%)	656 (131.2%)
Python regius	GH	2002	7000	6279 (89.7%)	9075 (129.6%)
Morelia amethistina	ID	2016	135	135 (100%)	174 (128.9%)
Amyda cartilaginea	ID	2010	25200	25104 (99.6%)	32060 (127.2%)
Kinixys homeana	GH	2003	340	165 (48.5%)	429 (126.2%)
Amyda cartilaginea	ID	2014	25200	25195 (100%)	30327 (120.3%)
Cuora amboinensis	ID	2006	18000	17694 (98.3%)	21482 (119.3%)
Varanus dumerilii	ID	2002	400	363 (90.8%)	476 (119%)
Amyda cartilaginea	ID	2015	25200	25200 (100%)	29882 (118.6%)
Leiopython albertisii	ID	2007	450	448 (99.6%)	521 (115.8%)
Epicrates cenchria	SR	2002	169	123 (72.8%)	195 (115.4%)
Python regius	GH	2006	7000	0 (0%)	8075 (115.4%)
Furcifer pardalis	MG	2005	2000	1855 (92.8%)	2304 (115.2%)
Cuora amboinensis	ID	2017	18000	15800 (87.8%)	20534 (114.1%)
Uroplatus ebenaui	MG	2016	150	124 (82.7%)	171 (114%)
Amyda cartilaginea	ID	2011	25200	24764 (98.3%)	28267 (112.2%)
Paleosuchus trigonatus	GY	2018	1000	994 (99.4%)	1120 (112%)
Phelsuma laticauda	MG	2005	2000	1834 (91.7%)	2220 (111%)
Chelonoidis carbonarius	GY	2004	704	548 (77.8%)	776 (110.2%)
Kinixys belliana	GH	2006	140	0 (0%)	154 (110%)
Epicrates cenchria	SR	2006	160	157 (98.1%)	176 (110%)
Chelonoidis carbonarius	GY	2018	704	662 (94%)	772 (109.7%)
Paleosuchus palpebrosus	GY	2004	500	381 (76.2%)	548 (109.6%)

Phelsuma laticauda	MG	2011	2000	1802 (90.1%)	2188 (109.4%)
Kinixys belliana	GH	2008	140	90 (64.3%)	150 (107.1%)
Furcifer lateralis	MG	2005	2000	1753 (87.7%)	2138 (106.9%)
Phelsuma quadriocellata	MG	2005	2000	1926 (96.3%)	2130 (106.5%)
Stigmochelys pardalis	TZ	2005	2350	2287 (97.3%)	2479 (105.5%)
Corallus caninus	GY	2001	880	828 (94.1%)	927 (105.3%)
Cuora amboinensis	ID	2009	18000	18000 (100%)	18960 (105.3%)
Phelsuma madagascariensis	MG	2005	2000	1926 (96.3%)	2098 (104.9%)
Cordylus rhodesianus	MZ	2002	1500	1020 (68%)	1570 (104.7%)
Morelia tracyae	ID	2019	27	26 (96.3%)	28 (103.7%)
Phelsuma kochi	MG	2016	1000	953 (95.3%)	1026 (102.6%)
Brookesia stumpffi	MG	2016	500	365 (73%)	510 (102%)
Heosemys spinosa	ID	2017	225	195 (86.7%)	229 (101.8%)
Kinixys erosa	GH	2006	120	0 (0%)	122 (101.7%)
Cuora amboinensis	ID	2010	18000	17965 (99.8%)	18239 (101.3%)
Furcifer pardalis	MG	2016	3000	2707 (90.2%)	3036 (101.2%)
Amyda cartilaginea	ID	2007	27000	26710 (98.9%)	27267 (101%)
Furcifer lateralis	MG	2011	2000	1909 (95.4%)	2019 (101%)
Furcifer oustaleti	MG	2005	2000	1806 (90.3%)	2013 (100.7%)
Furcifer pardalis	MG	2011	2000	1903 (95.2%)	2013 (100.7%)

Supplementary Table 3. Fixed effect coefficients for the pre- to post-quota trade volume model. Fixed effects should be interpreted as the association for the average species from the average exporter. Coeficients are on the standard deviation scale so the β_1 coefficient of -0.06 represent a decrease of 0.06 SD's relative to the reference level.

	**Median**	**Lower**	**Upper**	***pd*** **(%)**
Intercept *(reference level pre-quota trade)*	0.05	-0.18	0.34	62.75
β_1 Post-quota trade	-0.06	-0.34	0.24	61.95
β_2 Post-quota quota	0.82	0.45	1.12	100.00
β_3 Year *(reference level pre-quota trade)*	0.15	0.11	0.19	100.00
β_4 Year : Post-quota trade	-0.19	-0.23	-0.15	100.00
β_5 Year : Post-quota quota	-0.17	-0.22	-0.13	100.00

Supplementary Table 4. Change-point model summary of trends pre and post change-point. Coefficients are on the identity scale so a pre-change slope of 0.27 denotes a 0.27 increase in the number of updates per additional year of quota length.

	**Median**	**Lower**	**Upper**	**Pd (%)**
Pre-change slope	0.29	0.25	0.32	100.00
Post-change slope	-0.01	-0.11	0.10	56.90
**Contrast**	0.30	0.17	0.41	100.00

Supplementary Table 5. Description of each relationship between pre-quota volumes and post-quota quota levels or post-quota traded volumes. An increasing or decreasing trend direction is defined as a coefficient with a probability of direction >97.5%. Likewise, an increase or decrease in volume in the year quotas were implemented is where the pre to post contrast has a probability of direction >97.5%.

Relationship	Criteria	Description
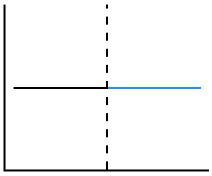	Pre-quota trend: **Uncertain**<br>Post-quota trend: **Uncertain**<br>Difference in volume at quota implementation: **Uncertain**	**No relationship**. Pre- and post-quota trends are neither increasing nor decreasing and there is no clear difference in absolute amounts.
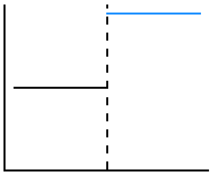	Pre-quota trend: **Uncertain**<br>Post-quota trend: **Uncertain**<br>Difference in volume at quota implementation: **Increase**	**Step increase**. Pre- and post-quota trends are neither increasing nor decreasing and there is a clear increase in absolute amounts relative to pre-quota.
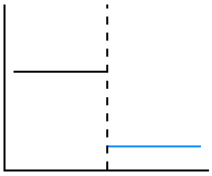	Pre-quota trend: **Uncertain**<br>Post-quota trend: **Uncertain**<br>Difference in volume at quota implementation: **Decrease**	**Step decrease**. Pre- and post-quota trends are neither increasing nor decreasing and there is a clear decrease in absolute amounts relative to pre-quota.
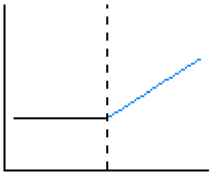	Pre-quota trend: **Uncertain**<br>Post-quota trend: **Increasing**<br>Difference in volume at quota implementation: **Uncertain**	**Increasing from a plateau.** Pre-quota trends are neither increasing nor decreasing, post-quota trends are increasing and there is no clear difference in absolute amounts.
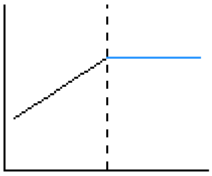	Pre-quota trend: **Increasing**<br>Post-quota trend: **Uncertain**<br>Difference in volume at quota implementation: **Uncertain**	**Increasing to a plateau.** Pre-quota trends are increasing, post-quota trends are neither increasing nor decreasing and there is no clear difference in absolute amounts.
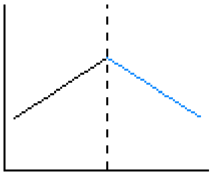	Pre-quota trend: **Increasing**<br>Post-quota trend: **Decreasing**<br>Difference in volume at quota implementation: **Uncertain**	**Trend shift**. Pre-quota trends are increasing, post-quota trends are decreasing and there is no clear difference in absolute amounts.
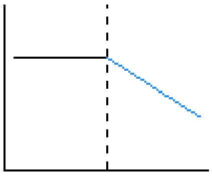	Pre-quota trend: **Uncertain**<br>Post-quota trend: **Decreasing**<br>Difference in volume at quota implementation: **Uncertain**	**Decreasing from a plateau.** Pre-quota trends are neither increasing nor decreasing, post-quota trends are decreasing and there is no clear difference in absolute amounts.

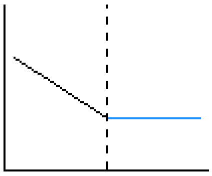	Pre-quota trend: **Decreasing**<br>Post-quota trend: **Uncertain**<br>Difference in volume at quota implementation: **Uncertain**	**Decreasing to a plateau.** Pre-quota trends are decreasing, post-quota trends are neither increasing nor decreasing and there is no clear difference in absolute amounts.
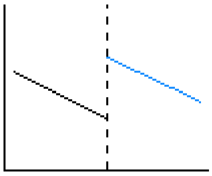	Pre-quota trend: **Decreasing**<br>Post-quota trend: **Decreasing**<br>Difference in volume at quota implementation: **Increase**	**Continuous temporal decrease, step increase.** Pre-quota trends are decreasing, post-quota trends are decreasing and there is an increase in absolute amounts relative to pre-quota.
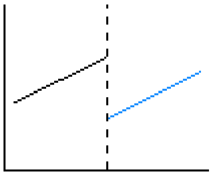	Pre-quota trend: **Increasing**<br>Post-quota trend: **Increasing**<br>Difference in volume at quota implementation: **Decrease**	**Continuous temporal increase, step decrease.** Pre-quota trends are increasing, post-quota trends are increasing and there is a decrease in absolute amounts relative to pre-quota.
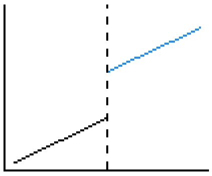	Pre-quota trend: **Increasing**<br>Post-quota trend: **Increasing**<br>Difference in volume at quota implementation: **Increase**	**Continuous temporal increase, step increase.** Pre-quota trends are increasing, post-quota trends are increasing and there is an increase in absolute amounts relative to pre-quota.
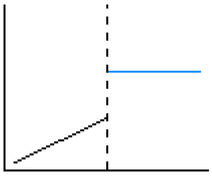	Pre-quota trend: **Increasing**<br>Post-quota trend: **Uncertain**<br>Difference in volume at quota implementation: **Increase**	**Increases to elevated plateau.** Pre-quota trends are increasing, post-quota trends are neither increasing or decreasing and there is an increase in absolute amounts relative to pre-quota.
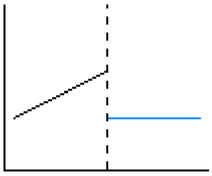	Pre-quota trend: **Increasing**<br>Post-quota trend: **Uncertain**<br>Difference in volume at quota implementation: **Decrease**	**Increases to reduced plateau.** Pre-quota trends are increasing, post-quota trends are neither increasing or decreasing and there is a decrease in absolute amounts relative to pre-quota.
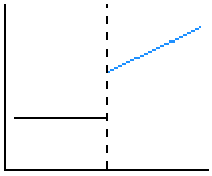	Pre-quota trend: **Uncertain**<br>Post-quota trend: **Increasing**<br>Difference in volume at quota implementation: **Increase**	**Post-quota trend and step increase.** Pre-quota trends are neither increasing or decreasing, post-quota trends are increasing and there is an increase in absolute amounts relative to pre-quota.
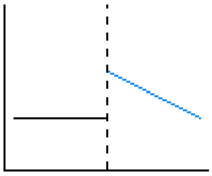	Pre-quota trend: **Uncertain**<br>Post-quota trend: **Decreasing**<br>Difference in volume at quota implementation: **Increase**	**Step increase, subsequent decline.** Pre-quota trends are neither increasing or decreasing, post-quota trends are decreasing and there is an increase in absolute amounts relative to pre-quota.
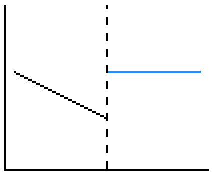	Pre-quota trend: **Decreasing**<br>Post-quota trend: **Uncertain**<br>Difference in volume at quota implementation: **Increase**	**Temporal decrease with elevated plateau.** Pre-quota trends are decreasing, post-quota trends are neither increasing or decreasing and there is an increase in absolute amounts relative to pre-quota

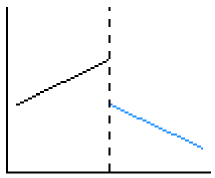	Pre-quota trend: **Increasing**<br>Post-quota trend: **Decreasing**<br>Difference in volume at quota implementation: **Decrease**	**Trend shift, increase to decrease, with step decrease.** Pre-quota trends are increasing, post-quota trends are decreasing and there is a decrease in absolute amounts relative to pre-quota.
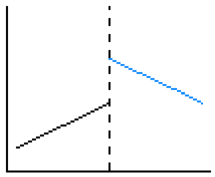	Pre-quota trend: **Increasing**<br>Post-quota trend: **Decreasing**<br>Difference in volume at quota implementation: **Increase**	**Trend shift, increase to decrease, with step increase.** Pre-quota trends are increasing, post-quota trends are decreasing and there is an increase in absolute amounts relative to pre-quota.

Supplementary Table 6. All species-exporter combinations where the species is assessed as likely threatened by the international wildlife trade reported as traded after 2015. The IUCN assessments shown correspond to the species status in the year it was traded, not necessarily their current status.

**Taxon**	Exporter	Year	Volume	IUCN Assessment
Carettochelys insculpta	ID	2018	5240	EN
Cyclanorbis senegalensis	CD	2019	15	VU
Cyclanorbis senegalensis	GH	2018	86	VU
Cyclanorbis senegalensis	GH	2021	235	VU
Cyclanorbis senegalensis	TG	2017	83	VU
Cyclanorbis senegalensis	TG	2018	9	VU
Cycloderma aubryi	CD	2017	50	VU
Graptemys pearlensis	US	2018	6	EN
Kinixys homeana	GH	2019	205	VU
Macrochelys temminckii	US	2016	43718	VU
Macrochelys temminckii	US	2017	35889	VU
Macrochelys temminckii	US	2018	36672	VU
Macrochelys temminckii	US	2019	29801	VU
Malaclemys terrapin	US	2017	72	NT
Ophiophagus hannah	ID	2021	174	VU
Ophiophagus hannah	MY	2016	10	VU
Ophiophagus hannah	MY	2017	16	VU
Ophiophagus hannah	MY	2018	2	VU
Ophiophagus hannah	MY	2019	1	VU
Ophiophagus hannah	MY	2020	2	VU
*Siebenrockiella crassicollis**	ID	2019	2349	VU
Trioceros serratus	CM	2019	10	VU
Trioceros serratus	CM	2020	78	VU
Trioceros serratus	CM	2021	260	VU
Uromastyx aegyptia	SD	2018	485	VU

***** *Siebenrockiella crassicollis* is quota managed annually by Indonesia 2003-2023, except in 2019 this may indicate the species was still managed in 2019 but the quota was just not submitted/published.
