# Supplementary material for: International wildlife trade quotas are characterized by high compliance and coverage but insufficient adaptive management
Source: Nat Ecol Evol. 2024 Sep 9;8(11):2048–57. doi: 10.1038/s41559-024-02531-4 (PMC11540902; doi:10.1038/s41559-024-02531-4)
Supplement: Supplementary file 2 — Reporting Summary [file 41559_2024_2531_MOESM2_ESM.pdf]

nature portfolio

Corresponding author(s): Oscar Morton

Last updated by author(s): Jul 24, 2024

Reporting Summary

Nature Portfolio wishes to improve the reproducibility of the work that we publish. This form provides structure for consistency and transparency in reporting. For further information on Nature Portfolio policies, see our Editorial Policies and the Editorial Policy Checklist.

Statistics

For all statistical analyses, confirm that the following items are present in the figure legend, table legend, main text, or Methods section.

n/a	Confirmed	
☐	☒	The exact sample size (*n*) for each experimental group/condition, given as a discrete number and unit of measurement
☒	☐	A statement on whether measurements were taken from distinct samples or whether the same sample was measured repeatedly
☒	☐	The statistical test(s) used AND whether they are one- or two-sided<br>*Only common tests should be described solely by name; describe more complex techniques in the Methods section.*
☐	☒	A description of all covariates tested
☐	☒	A description of any assumptions or corrections, such as tests of normality and adjustment for multiple comparisons
☐	☒	A full description of the statistical parameters including central tendency (e.g. means) or other basic estimates (e.g. regression coefficient) AND variation (e.g. standard deviation) or associated estimates of uncertainty (e.g. confidence intervals)
☒	☐	For null hypothesis testing, the test statistic (e.g. *F*, *t*, *r*) with confidence intervals, effect sizes, degrees of freedom and *P* value noted<br>*Give P values as exact values whenever suitable.*
☐	☒	For Bayesian analysis, information on the choice of priors and Markov chain Monte Carlo settings
☐	☒	For hierarchical and complex designs, identification of the appropriate level for tests and full reporting of outcomes
☒	☐	Estimates of effect sizes (e.g. Cohen's *d*, Pearson's *r*), indicating how they were calculated

Our web collection on statistics for biologists contains articles on many of the points above.

Software and code

Policy information about availability of computer code

Data collection	Data was directly downloaded from publicly accessible sites that are clearly linked to in the manuscript methods.
Data analysis	All analysis was completed using R version 4.3.1<br>Key packages used were:<br>tidyverse 2.0.0<br>rredlist 0.7.1<br>brms 2.20.4<br>tidybayes 3.0.6<br>ggpubr 0.6.0<br>viridis 0.6.4<br><br>All code is publically available from github with the link clearly given in the manuscript.

For manuscripts utilizing custom algorithms or software that are central to the research but not yet described in published literature, software must be made available to editors and reviewers. We strongly encourage code deposition in a community repository (e.g. GitHub). See the Nature Portfolio guidelines for submitting code & software for further information.

Data

Policy information about availability of data

All manuscripts must include a data availability statement. This statement should provide the following information, where applicable:

- Accession codes, unique identifiers, or web links for publicly available datasets
- A description of any restrictions on data availability
- For clinical datasets or third party data, please ensure that the statement adheres to our policy

All data used in this study is publicly accessible and referenced in the main manuscript, the processed data used in the final analyses is available from https://github.com/OMorton/CITES_Quotas.

Research involving human participants, their data, or biological material

Policy information about studies with human participants or human data. See also policy information about sex, gender (identity/presentation), and sexual orientation and race, ethnicity and racism.

Reporting on sex and gender	NA
Reporting on race, ethnicity, or other socially relevant groupings	NA
Population characteristics	NA
Recruitment	NA
Ethics oversight	NA

Note that full information on the approval of the study protocol must also be provided in the manuscript.

Field-specific reporting

Please select the one below that is the best fit for your research. If you are not sure, read the appropriate sections before making your selection.

☐ Life sciences ☐ Behavioural & social sciences ☒ Ecological, evolutionary & environmental sciences

For a reference copy of the document with all sections, see nature.com/documents/nr-reporting-summary-flat.pdf

Ecological, evolutionary & environmental sciences study design

All studies must disclose on these points even when the disclosure is negative.

Study description	This study analyzed the diversity of taxa and trade types covered by current international trade quotas. We then further assessed national compliance with trade quotas using reported trade data. We analyzed national trade volumes pre- and post-quota implementation to assess how the setting of a quota affected trade volumes.
Research sample	Records of all specified quotas for internationally traded CITES-listed species, access from https://speciesplus.net/. This was augmented with the international trade data taken from https://trade.cites.org/. The combined data set represents all international trade in listed species and their compliance with internationally set quotas between 1997 and 2021. The data is representative of all legal international trade in CITES listed species to or from Parties, not of all trade.
Sampling strategy	No sample size calculations were used. All available quota data was used.
Data collection	Data was not collected by the authors data was collected by the UNEP-WCMC and the Parties themselves. OM downloaded and processed the data in R.
Timing and spatial scale	The data was "collected" from the online repositories in Autumn 2023. We used the 2023 version of the trade data, which will be the most recent and up to data until at least summer 2024. We used the maximum data range that can be appropriately used, starting in 1997 which was when the first international trade quotas were established and ending in 2021 which is the most recent year traded data should be used due to delayed reporting by some Parties.
Data exclusions	In the methods we clearly detail the limited circumstances where analyses focused on a subset of quotas. Namely these include the analysis limited to only live quotas (e.g. not skins, leather products etc.) other quota types were excluded as they cannot be objectively quantified.
Reproducibility	This was not an experiment. However we did do an additional robustness analysis using the importer reported data (main text used

Reproducibility	the exporter reported trade data). Due to differing reporter obligations these data sets are not expected to be identical, yet our results using either dataset lead to the same conclusions.
Randomization	NA
Blinding	NA

Did the study involve field work? ☐ Yes ☒ No

Reporting for specific materials, systems and methods

We require information from authors about some types of materials, experimental systems and methods used in many studies. Here, indicate whether each material, system or method listed is relevant to your study. If you are not sure if a list item applies to your research, read the appropriate section before selecting a response.

Materials & experimental systems

n/a	Involved in the study	
☒	☐	Antibodies
☒	☐	Eukaryotic cell lines
☒	☐	Palaeontology and archaeology
☒	☐	Animals and other organisms
☒	☐	Clinical data
☒	☐	Dual use research of concern
☒	☐	Plants

Methods

n/a	Involved in the study	
☒	☐	ChIP-seq
☒	☐	Flow cytometry
☒	☐	MRI-based neuroimaging

Plants

Seed stocks	NA
Novel plant genotypes	NA
Authentication	NA
